# Histone H3 Lysine 56 Acetylation Is Required for Formation of Normal Levels of Meiotic DNA Breaks in *S. cerevisiae*

**DOI:** 10.3389/fcell.2019.00364

**Published:** 2020-01-10

**Authors:** Zsolt Karányi, Lilla Hornyák, Lóránt Székvölgyi

**Affiliations:** ^1^MTA-DE Momentum Genome Architecture and Recombination Research Group, Department of Biochemistry and Molecular Biology, Faculty of Medicine, University of Debrecen, Debrecen, Hungary; ^2^Department of Internal Medicine, Faculty of Medicine, University of Debrecen, Debrecen, Hungary

**Keywords:** recombination, DNA break, meiosis, histone modification, H3K56 acetylation

## Abstract

Meiotic recombination is initiated by Spo11-catalyzed DNA double-strand breaks (DSBs) that are promoted by histone modifications and histone modifying enzymes. Herein we investigated the role of histone H3 lysine 56 acetylation (H3K56ac) located near the entry/exit points of the DNA in the globular H3 domain. We generated a series of mutant cells (*asf1*Δ, *rtt109*Δ, *hst3/4*Δ, and H3K56A) in which the endogenous level of H3K56ac was manipulated and tracked during meiotic growth. We show that complete loss or increased abundance of H3K56ac in these mutants allows timely entry into meiosis and sporulation and does not impair S phase progression, first and second meiotic cell divisions, and spore viability. In the *asf1*Δ, *rtt109*Δ, *hst3/4*Δ mutants, DSBs and crossovers form normal levels with a short (60-min) delay at the *HIS4-LEU2* artificial recombination hotspot, however, DSB formation shows a ∼threefold decrease in the H3K56A mutant at the natural *BUD23-ARE1* hotspot. The latter DSB phenotype, showing significant DSB reduction in the H3K56A mutant, was also observed at DSB sites using genome-wide mapping of Rfa1-coated single-stranded DNA flanking DSBs (RPA ChIP). Parallel mapping of H3K56-acetylated histones in wild type cells revealed strong depletion of the H3K56ac ChIP signal over Spo11-oligo DSBs, albeit most H3K56-acetylated histones were enriched adjacent to the identified RPA ChIP binding sites. Taken together, these associations demonstrate a prominent role of H3 lysine 56 acetylation in the formation of DNA breaks within recombination hotspot regions.

## Introduction

Meiosis is a cellular differentiation process which is accompanied by high levels of recombination between the homologous chromosomes, initiated by DNA double-strand breaks (DSBs) catalyzed by Spo11 and accessory factors (Keeney et al., 1997; Székvölgyi and Nicolas, 2010; Székvölgyi et al., 2015). In *S. cerevisiae*, meiotic DSBs are controlled by the elements of chromatin structure. This involves a complex interplay between DNA sequence composition, local chromatin status, nucleosome occupancy, and transcription factor binding (Pan et al., 2011). Among these factors, histone modifications represent an important layer which has only recently been explored in detail. The most well-characterized histone modification is H3K4me3 that appears to be essential for recombination sites in most organisms. In the current model, H3K4me3 is deposited by Set1C and becomes recognized by Spp1 (the PHD finger subunit of Set1C), which leads to tethering of DSB sites to the chromosome axis that undergo Spo11-mediated cleavage (Acquaviva et al., 2013; Sommermeyer et al., 2013; Karányi et al., 2018). A similar mechanism has been proposed in mammals involving the meiosis-specific H3K4 methylase Prdm9 (Baudat et al., 2010; Parpanov et al., 2010), however, CXXC1 (the yeast ortholog of Spp1) is apparently not essential for the association of H3K4 tri-methylated recombination sites with the DSB machinery (Tian et al., 2018).

The widely localized H3K4me3 mark has the virtue of initiating recombination at numerous places in the genome, however, other histone modifications are also needed to keep recombination flexible for the diversity of recombinant haplotypes (Szekvolgyi and Nicolas, 2010). These “alternative” pathways remain to be clarified to better understand the plasticity of crossover patterning. Most chemical modifications are concentrated at the N-termini of histones and are not expected to alter the structure of nucleosomes (White et al., 2001; Biswas et al., 2011). However, modifications of histone core domains can directly change nucleosome structure, which is well-established biochemically (Luger et al., 1997; Biswas et al., 2011) but its functional relevance is less understood.

In the current study, we focused on the role of histone H3 lysine 56 acetylation located near the entry/exit points of the DNA in the globular H3 domain, predicted to destabilize the histone/DNA contact (Buning and Van Noort, 2010; Simon et al., 2011). H3K56ac is a transient chromatin signal showing rapid turnover and is closely linked to DNA replication and histone eviction during transcription (Rufiange et al., 2007; Watanabe et al., 2013). Functional studies in mitotically proliferating yeast cells revealed that H3K56ac enables the assembly and disassembly of nucleosomes during DNA synthesis and upon transcriptional activation. Furthermore, the histone chaperone Asf1 [carrying the H3K56-specific acetyltransferase Rtt109 (Abshiru et al., 2013)] and the histone residue H3K56 were found to be necessary for meiotic S phase progression and for the development of reproductive capacity in yeast and mouse models (Recht et al., 2006; Govin et al., 2010; Messiaen et al., 2016). However, the mechanism of action of H3K56 acetylation has not been fully elucidated and remains to be clarified. Therefore, we applied a functional approach in meiotic *S cerevisiae* cells to modify the natural levels of H3K56ac to study the biochemical phases of meiosis. Our results demonstrate that H3K56ac is necessary for formation of normal levels of DSBs within recombination hotspot regions.

## Methods

All methods are available as Supplementary Material.

## Results

**FIGURE 1:**
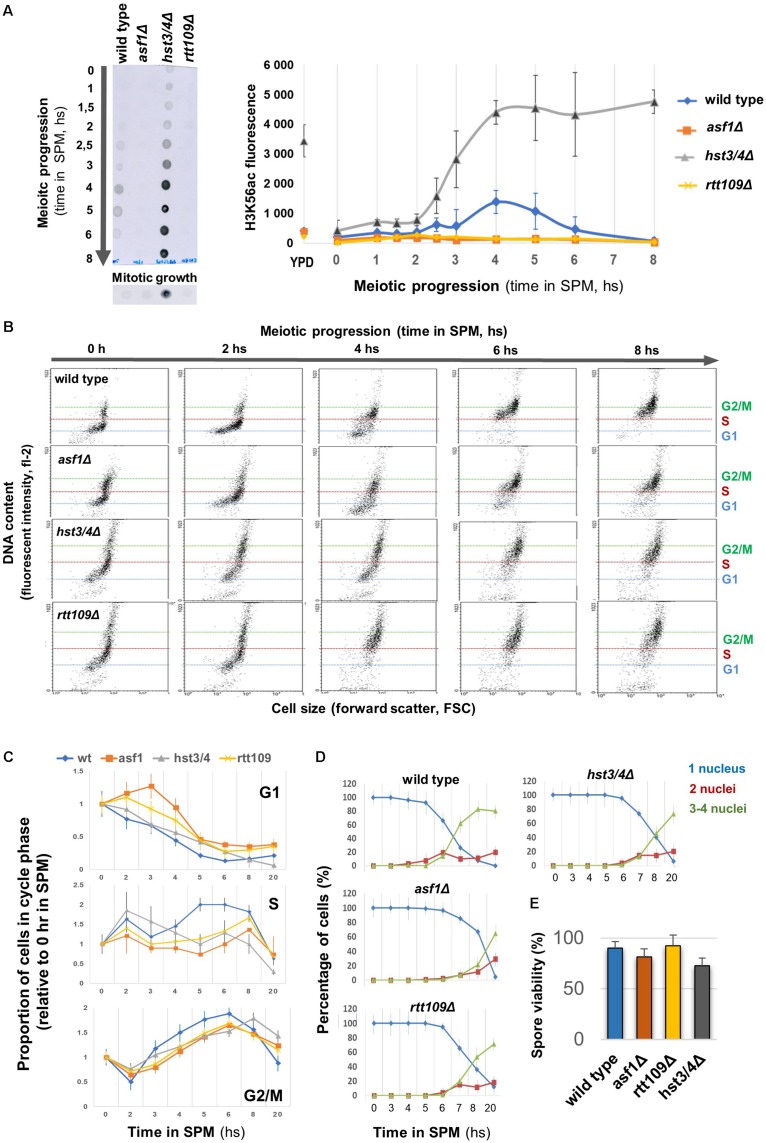
Different H3K56ac levels allow normal meiotic progression and sporulation. **(A)** Left: Representative dot blot showing H3K56ac levels in wild type, *asf1*Δ, *rtt109*Δ, and *hst3/4*Δ cells. Right: Quantification of H3K56ac levels. YPD: mitotic growth. **(B)** Representative FACS profile of sporulating strains, showing DNA content in terms of cell size. G1, S, G2/M phases are indicated. **(C)** Proportion of cells in G1, S, and G2/M based on meiotic FACS profiles. Data were normalized to 0 h in SPM. **(D)** Sporulation efficiency. Nuclei were stained with DAPI and cells were scored for nucleus count. **(E)** Spore viability (50 tetrads per strain were counted). Panels **(A,C–E)** show the mean of three independent replicas. Error bars: SEM.

We generated mutant cells in which the endogenous level of H3K56ac was modified and tracked during meiotic growth (Figure 1A). In wild-type cells, the level of H3K56ac reached a peak during DSB formation (at ∼4–5 h in SPM) and then rapidly dropped, similar to earlier western blot results (Recht et al., 2006). The H3K56ac signal disappeared almost completely from *asf1*Δ and *rtt109*Δ deletion mutants during the meiotic time course (∼fourfold reduction compared to wild type), in agreement with the crucial role of Asf1 and Rtt109 in the deposition of this epigenetic tag (Tsubota et al., 2007). The K56ac signal increased and remained high in the *hst3/4*Δ double-mutant that prevents deacetylation of H3K56 (Celic et al., 2006). In mitotic growth conditions, we could not detect an H3K56 acetylation signal, except for the *hst3/4*Δ mutant (Figure 1A), in which H3K56ac persists throughout the cell cycle (Celic et al., 2006). This is in accordance with the transient nature of H3K56 acetylation in asynchronously proliferating mitotic cells, mainly associated with newly replicated chromatin regions (Simoneau et al., 2015).

The correlation between H3K56ac dynamics and the phase of meiotic DSBs prompted us to analyze the progress of S phase, DSB formation, sporulation efficiency, and spore viability in cells with various H3K56ac levels. FACS analysis revealed a 30–60 min delay in G1/S phase progression in mutant cells, however, all mutants reached the G2/M phase within 6 h in SPM (Figures 1B,C). We then performed a meiotic time course up to 20 h. The results reported in Figures 1D,E show that sporulation efficiencies and spore viabilities do not differ between mutant and wild-type cells (less than 1.5-fold change was observed in the number of tetrads and viable spores). We conclude that absence or elevated levels of H3K56ac is dispensable for meiotic S phase progression, MI/MII cell divisions, and spore viability.

**FIGURE 2:**
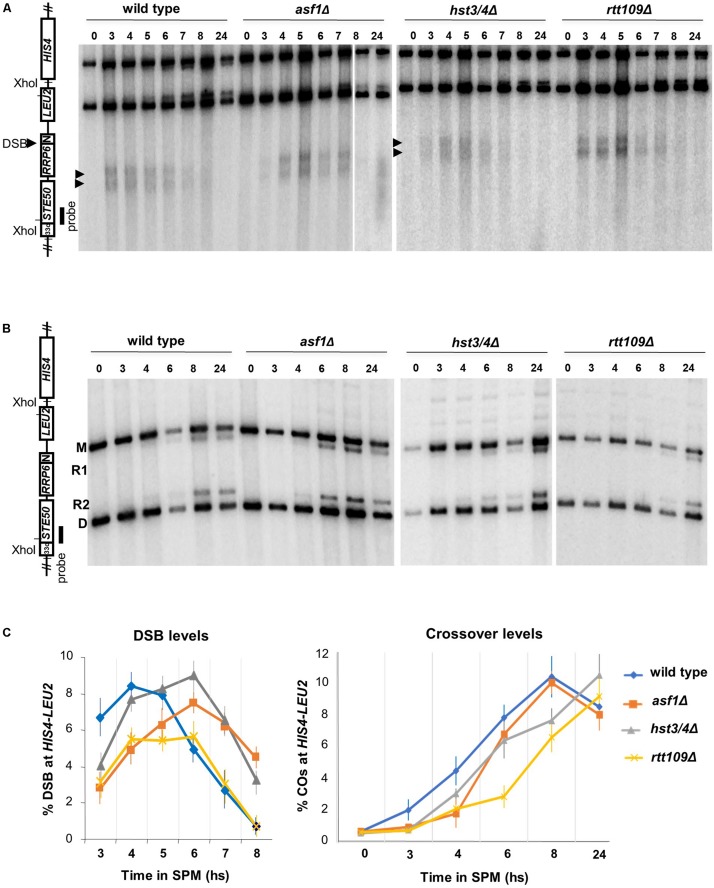
Southern blot analysis of DSB and crossover formation at the *HIS4-LEU2* hotspot. **(A)** Representative blot showing *Xho*I-digested DNA from wild-type, *asf1*Δ, *hst3/4*Δ, and *rtt109*Δ cells. **(B)** Representative blot showing recombinant products (R1, R2) and parental bands (M, D) at *HIS4-LEU2*. **(C)** Quantification of DSB levels (% DSB signal/total lane signal) and recombination frequencies [% (R1 + R2) signal/total lane signal]. Mean values are from three independent replicas. Error bars: SEM.

We next analyzed whether DSBs were affected in mutants (with Sae2^+^/Rad50^+^ background). DSB levels were monitored at the *HIS4*-*LEU2* hotspot (Xu and Kleckner, 1995) using Southern blot hybridization (Figure 2A). All mutants produced comparable DSB levels relative to wild-type (between 6 and 8% based on the global maxima of the curves), with a ∼60 min delay in *asf1*Δ and *hst3/4*Δ mutants (Figure 2C). The delay of DNA breaks in *asf1*Δ and *hst3/4*Δ cells suggests that DSBs appear and disappear with a slightly modified kinetics in these mutants. Maximal DSB reduction was observed in the *rtt109*Δ strain (1.5-fold reduction). Importantly, meiotic DSBs detected in the mutants were highly recombinogenic (R1/R2 recombinants are highlighted in Figure 2B) forming wild-type levels of crossovers (∼10–11%, Figure 2C). These results suggest that DSBs detected at *HIS4*-*LEU2* were properly processed to form mature recombination products.

**FIGURE 3:**
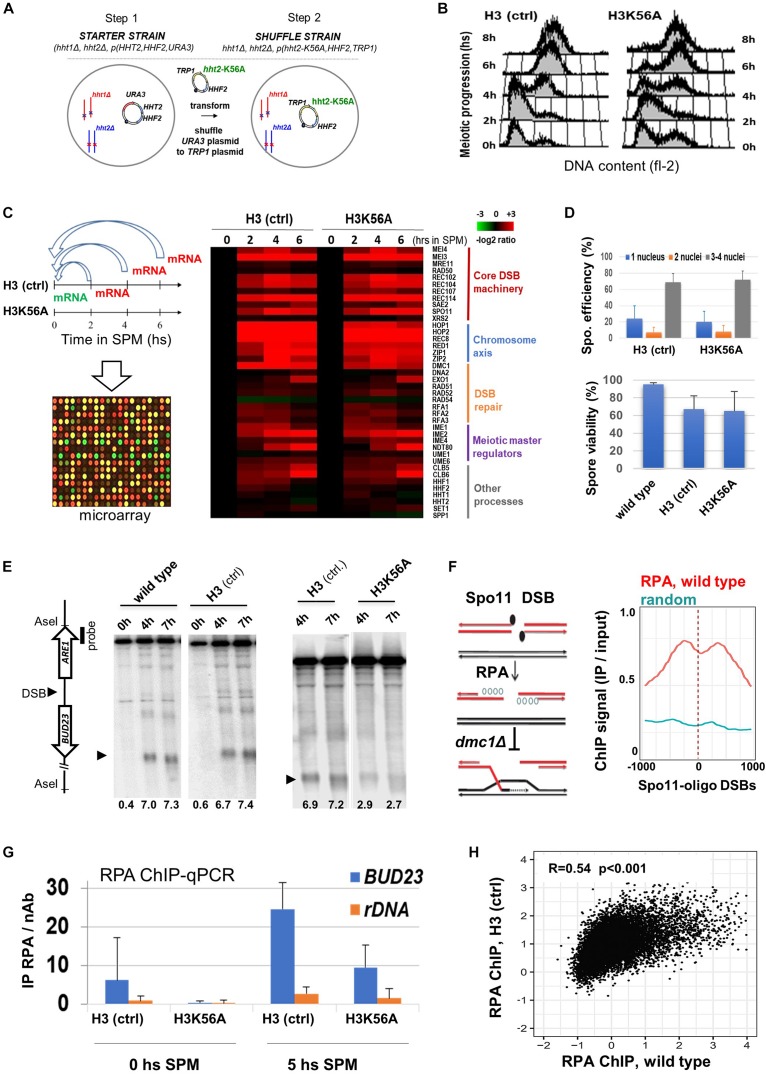
Sporulation and meiotic DSB formation in a H3K56A mutant. **(A)** H3K56A cells were constructed by plasmid shuffling. **(B)** S phase progression in H3K56A and H3 (ctrl) cells measured by FACS. **(C)** Meiotic transcriptome dynamics in H3 (ctrl) and H3K56A strains. Left: Relative mRNA levels as measured by transcriptome microarrays at 2, 4, 6 h in SPM compared to 0 h in SPM. Right*:* Heatmap highlighting a selection of “core” genes that govern the process meiosis. The warmer the color, the greater the degree of induction. Full details are provided in Supplementary Table S1. **(D)** Sporulation efficiency and spore viability of H3K56A, H3 (ctrl), and wild-type strains. Error bars: SEM. **(E)** DSBs within *BUD23-ARE1*. Representative blot shows *Ase*I-digested gDNA from wild type, H3 (ctrl) and H3K56A cells. DSB levels (below the blots): % DSB signal/total lane signal. **(F)** Left: Outline of the RPA ChIP approach, showing resected ssDNAs covered by RPA, which accumulates in *dmc1*Δ cells. Right: RPA enrichment over Spo11-oligo DSB sites and random sites in wild-type SK1 cells. Wild-type RPA data are from Borde et al. (2009). **(G)** ChIP-qPCR analysis of RPA enrichment at 0 and 5 h in SPM in the *BUD23* hotspot and the *rDNA* negative control site (known to lack DSBs). Error bars: SEM. **(H)** Correlation of RPA ChIP profiles in H3 (ctrl) and wild-type cells. R: Pearson correlation coefficient.

To obtain mechanistic insights whether the modifiable H3K56 residue influences DSB formation, we constructed a plasmid shuffle system that allows the expression of wild-type H3K56 (H3 ctrl) and unmodifiable H3K56A as the only source of histone H3 (Figure 3A). Like wild-type cells, H3K56A mutants progressed synchronously through the meiotic S phase (Figure 3B) and showed normal transcriptome dynamics with no difference in gene expression during meiosis (Figure 3C and Supplementary Table S1). “Core” DSB, chromosome axis, and repair genes were properly transcribed in H3K56A cells, including *SPO11* and *RFA1* (Supplementary Figure S1), which were both essential for subsequent meiotic DSB mapping using RPA ChIP (see later). Sporulation efficiency and spore viability of the H3K56A mutant was not different from H3 (ctrl) expressing wild-type H3 (Figure 3D).

Next, we analyzed DSB formation in H3 *dmc1*Δ and H3K56A *dmc1*Δ cells that accumulate unrepaired DSB ends. DSB frequencies were determined by Southern blot within the natural hotspot region *BUD23-ARE1* (Figure 3E). Quantification of DSBs in wild-type and H3 (ctrl) strains confirmed the correct location, timing and frequency of DSBs in plasmid shuffle cells, demonstrating that our system accurately reports DSB formation. Importantly, a threefold reduction of DSB levels was observed in the H3K56A mutant, which was subsequently confirmed by an independent RPA ChIP method capturing Rfa1-covered ssDNA flanking Spo11-oligo DSBs (Figures 3F–H). In plasmid shuffle cells, RPA levels increased in the *BUD23* hotspot region by 5 h in SPM when DSBs are formed, and H3K56A mutants showed a ∼threefold decrease in RPA levels relative to H3 (ctrl) (Figure 3G), consistent with our Southern blot results (Figure 3E). In addition, the RPA profiles of H3 (ctrl) and wild-type cells (Borde et al., 2009) were positively correlated (Figure 3H), whereas the RPA binding sites did not correlate with the binding of Mcm2-7 replicative helicase that marks meiotic DNA replication (Supplementary Figure S2). These results collectively demonstrate that RPA enrichment is an adequate indicator of meiotic DSB locations and frequencies.

**FIGURE 4:**
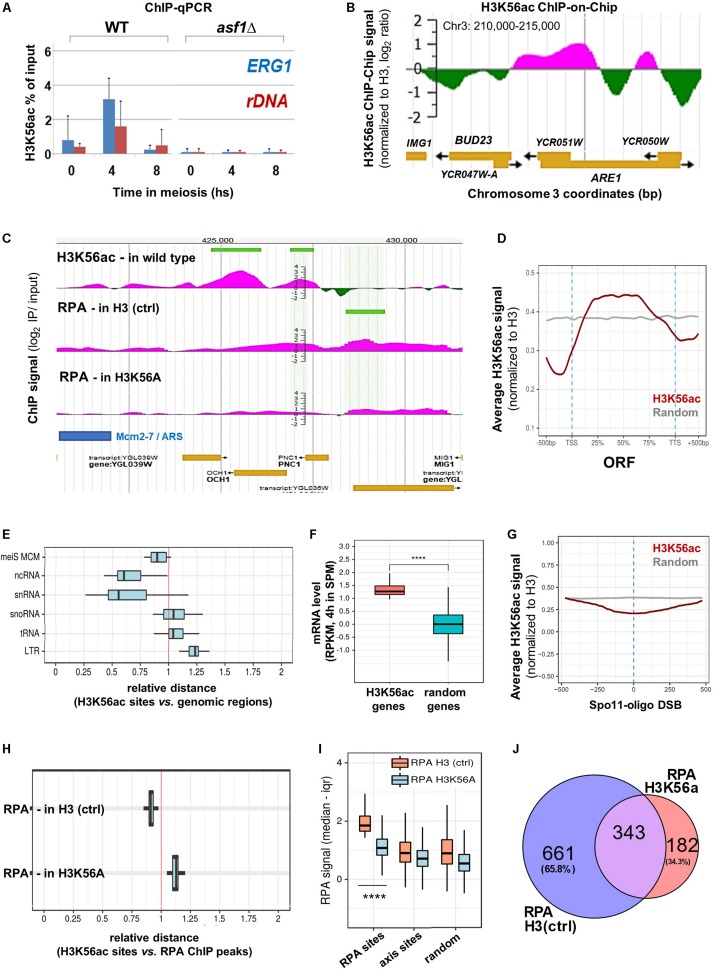
Genome-wide mapping of H3K56ac and RPA. **(A)** H3K56ac capture using an anti-H3K56ac antibody. H3 (C-ter) ChIP was performed to normalize to H3 occupancy. ChIP-qPCR enrichments were measured at *ERG1* and *rDNA*. Error bars: SEM. **(B)** H3K56ac signal detected by microarray (at 5 h in SPM). IP/input ratios were normalized to H3 occupancy (Borde et al., 2009). **(C)** Genome browser snapshot showing the distribution of H3K56ac in wild type cells and RPA enrichment in H3 (ctrl) and H3K56A cells. Peaks are highlighted by horizontal green lines. **(D)** Metagene profile of H3K56ac over protein-coding genes. Red curve shows the median H3K56ac signal. Gray curve: random signal. **(E)** Relative distance of H3K56ac peaks from various genomic regions. Vertical red line: random distances. Shift toward the left or right sectors: clustering or repulsion between H3K56ac peaks and the studied genomic elements. Mei MCM: meiotic replication origins (Blitzblau et al., 2012). **(F)** Genes associated with high H3K56ac levels show increased mRNA expression compared to random genes. Statistical significance: *p* < 0.0001 (Mann–Whitney *U* test). RNA-seq data are from Brar et al. (2012). **(G)** Depletion of H3K56ac over Spo11-oligo DSBs (Mohibullah and Keeney, 2017). Gray line: random signal. **(H)** Relative distance of H3K56ac peaks from RPA peaks in H3 (ctrl) and H3K56A cells. Vertical red line: random distance. **(I)** Distribution of RPA ChIP signal at RPA binding sites, axis sites (Mer2; Panizza et al., 2011), and random sites in H3 (ctrl) and H3K56A cells. Statistical significance: *p* < 0.0001 (Mann–Whitney *U* test). **(J)** Overlap of RPA peaks in H3 (ctrl) and H3K56A cells.

Notably, the *BUD23* and *ERG1* hotspot regions were flanked by H3K56ac at the time of DSB formation as measured by H3K56ac ChIP-qPCR and ChIP-chip (Figures 4A,B). We note that the H3K56ac ChIP signal was undetectable in *asf1*Δ cells that are deficient in H3K56 acetylation (Figure 1A), confirming the specificity of our ChIP assay. Furthermore, the H3K56ac signal was also depleted in wild-type cells within the recombinationally cold *rDNA* region (Figure 4A). The same associations were observed at the genomic scale (Figure 4C and *JBrowse* link), suggesting that the presence of H3K56 residue and/or deposition of H3K56-acetylated nucleosomes near DSB hotspots is required for complete DSB formation. Our H3K56ac ChIP results show that H3K56 acetylation is preferentially associated with protein-coding ORFs (Figure 4D) and is depleted from promoters and transcription termination sites [in line with published ChIP-qPCR data in mitotic cells (Schneider et al., 2006)]. The relative distance of H3K56ac from several genomic elements showed a clear non-random distribution (Figure 4E) as H3K56ac peaks were preferentially associated with Mcm2-7 helicase binding sites (Blitzblau et al., 2012), ncRNA and snRNA genes, but were further away from snoRNAs, tRNAs, and LTR retrotransposons compared to a computer-randomized chromosomal distribution (vertical red line in the figure). The proximity of H3K56ac peaks to Mcm2-7 binding sites agrees with the suggested role of H3K56 acetylation in marking nascent (replicating) chromatin upon replicative and repair DNA synthesis (Yu et al., 2012). Regarding meiotic gene expression (Figure 4F), protein-coding genes enriched in H3K56ac showed significantly higher mRNA expression levels in meiosis (at 4 h in SPM) than random genes [*p* < 0.0001; RNA-seq data are from Brar et al. (2012)]. Increased expression of H3K56-acetylated genes supports previous results in mitotic cells (Schneider et al., 2006; Williams et al., 2008), indicating that H3K56ac is an active chromatin mark associated with transcription.

Regarding meiotic DSB sites, chromosomal distribution of the H3K56ac signal showed a depletion over Spo11-oligo DSBs (Figure 4G), which precisely mark recombination hotspots. However, genomic positions of H3K56-acetylated histones were preferentially enriched adjacent to the identified RPA binding sites relative to random distance (Figure 4H, RPA H3 ctrl). Importantly, the RPA ChIP signal detected in the H3K56A mutant was strongly reduced at the RPA sites identified in the H3 (ctrl) strain (*p* < 0.0001, Mann–Whitney *U* test), as opposed to chromosome axis sites (Mer2-tagged; Panizza et al., 2011) and randomly selected sites that did not differ between control and mutant strains (Figure 4I). The preferential decrease of RPA ChIP signal at RPA binding sites in the H3K56A mutant demonstrates the specificity of H3K56A substitution mutation for recombination hotspot regions, accumulating hyper-resected ssDNA in the vicinity of DSBs. The specific decrease in the number of RPA peaks in the H3K56A mutant is highlighted in Figure 4J. This Venn diagram analysis shows that 65.8% of RPA peaks were eliminated by the H3K56A mutation (661 sites out of 1004), whereas 343 RPA sites were not affected or the signal was even increased (182 peaks). The latter RPA binding sites may represent unscheduled DSBs that are not related to H3K56 acetylation, which is clearly apparent from the increased relative distance between H3K56ac histones and RPA ChIP peaks detected in the H3K56A mutant (Figure 4H, RPA-H3K56A).

## Discussion

The above functional results highlight the association of H3K56 acetylation and meiotic DSB formation, suggesting that H3K56-acetylated histones are required to produce normal levels of DSBs within recombination hotspot regions. Nevertheless, the exact molecular mechanism underlying the DSB-promoting effect of H3K56 acetylation has yet to be clarified. The cause of the observed differences between the two mutant systems (histone modifying enzyme deletion vs. histone mutation) is currently not known. We obtained complementary results that are fully consistent with recent data identifying differences in the RNAPII binding profile of *rtt109*Δ and H3K56R mutants (Topal et al., 2019). Based on the genome-scale analysis of H3K56A cells, we propose that lack of H3K56 acetylation affects the stability or turnover rate of the well-positioned first nucleosomes flanking DSB sites (consistent with Kaplan et al. (2008)], and this may reduce the efficiency of Spo11-catalyzed DNA cleavage. Moreover, the absence of H3K56ac mark could reduce the rate of DSB end dissociation from nucleosomes flanking DSB sites, impeding the timely resection and processing of DSB ends. Alternatively, the H3K56A mutation may exert its effect indirectly on DSB formation, however, this is probably independent of changes in transcription since no differential gene expression was detected in the H3K56A mutant. A further possibility could be that H3K56 acetylation promotes the interaction of H3K4me3, Spp1, and Mer2 during the loop tethering process. The recently identified epistatic relationship of H3K56ac and H3K4me3 supports this hypothesis and seems particularly important in this regard (Voichek et al., 2018). H3K56ac was found to act upstream of Set1C and H3K4 methylation, generating complementary H3K56ac/H3K4me3 histone modification patterns along newly replicated chromatin. These functional relationships could readily allow close cooperation between H3K56ac and H3K4me3 during meiotic recombination, especially because newly replicated chromatin is rich in H3K56ac and DNA replication is mechanically coupled to meiotic DSB formation (Murakami and Keeney, 2014). We assume that these complex spatial interactions occur in the context of 3D chromatin structure. This could be detected by chromosome conformation capture methods (Dekker et al., 2017). Future use of these C-based approaches, together with mutant analyses, is expected to provide a deeper understanding of meiotic DSB formation with regards to the role of H3K4 methylation, H3K56 acetylation, and other potentially relevant histone modifications.

## Data Availability Statement

Datasets generated for this study can be accessed in Supplementary Table S1 and via JBrowse (login: h3k56ac, password: Mozaic4, http://geneart.med.unideb.hu/pub/h3k56ac). Raw data are availabe at GEO (GSE37487).

## Author Contributions

ZK and LH analyzed the genomic data. LS performed the experiments and wrote the manuscript.

## Conflict of Interest

The authors declare that the research was conducted in the absence of any commercial or financial relationships that could be construed as a potential conflict of interest.
