# Supplementary material for: Histone H3 Lysine 56 Acetylation Is Required for Formation of Normal Levels of Meiotic DNA Breaks in S. cerevisiae
Source: Front Cell Dev Biol. 2020 Jan 10;7:364. doi: 10.3389/fcell.2019.00364 (PMC6970188; doi:10.3389/fcell.2019.00364)
Supplement: Supplementary file 1 [file Data_Sheet_1.pdf]

Supplementary Material

Supplementary Figures

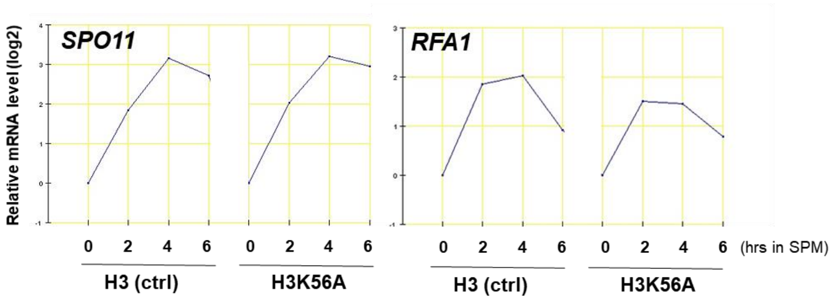

Supplementary Figure S1. Dynamics of *SPO11* and *RFA1* messenger RNAs expression during a meiotic time course (related to Figure 3). *x axis*: Strains (H3 (ctrl) and H3K56A) and meiotic time points (0/2//4/6 hs in SPM). *y axis*: Relative mRNA levels compared to the start of the sporulation process (log_2 ratios) as measured by transcriptome microarrays. There is no difference between the mRNA induction of *SPO11* and *RFA1* in the H3 (ctrl) and H3K56A strains. Spo11 DSBs were subsequently mapped by RPA (Rfa1) ChIP (see Figure 3F).

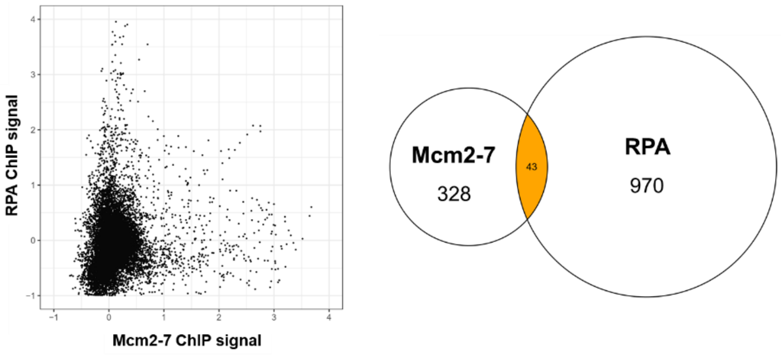

Supplementary Figure S2. Lack of correlation between the chromosomal association of RPA and meiotic DNA replication (related to Figure 3). *left:* Correlation plot showing the association of RPA and Mcm2-7 ChIP profiles in meiosis. The two variables are not correlated. *right:* Venn diagram showing the overlap of Mcm2-7 binding sites and RPA binding sites. RPA and Mcm2-7 ChIP-chip data are from (1,2).

Extended Methods

Yeast strain construction

The strains used in this study are from the SK1 background. The *asf1Δ*::KanMX, *rtt109Δ*::*TRP1*, and *hst3Δ*::KanMX *hst4Δ*::HphMX mutations were introduced by transformation with PCR-amplified deletion cassettes from the EUROFAN II gene deletion library, and crossing. All deletions were verified by Southern blotting and PCR. Plasmid shuffle strains were constructed by transforming isogenic *hht1Δ*::HphMX *hht2Δ*::KanMX pARS-CEN(*HHT2*, *HHF2-URA3*) and *hht1Δ*::HphMX *hht2Δ*::KanMX *dmc1Δ*::*LEU2* pARS-CEN(*HHT2*, *HHF2-URA3*) strains with a pARS-CEN(*hht2*-K56A, *HHF2-TRP1*) plasmid. The *hht2*-K56A mutation was introduced into the above *TRP1* plasmid by QuikChange II PCR mutagenesis (Agilent). The *URA3* plasmid was segregated by overnight growth in rich media (YPD). Single colonies were grown in YPD that were subsequently replicated onto -ura, -trp drop-out plates to identify trp+, ura- colonies. The presence of histone H3K56A point mutation was verified by Sanger sequencing.

Measurement of meiotic progression, sporulation efficiency and spore viability

1 ml of cells were harvested from the meiotic time courses at the indicated time points and were immediately fixed in 70% ethanol. Meiotic S phase progression was monitored by flow cytometry (FacsCalibur, Becton-Dickinson, USA). Sporulation efficiency was assessed by fluorescent microscopic analysis of 4'-6-diamidino-2-phenylindole (DAPI) stained nuclei. Spore viability was calculated by dissection of full tetrads (MSM 400 microdissector, Singer Instruments, UK) and determining the ratio of viable spores. We regarded >2-fold reduction in spore viability as significant change in fertility.

Dot blot

1.5 ml of sporulating cell culture was harvested and pellets were immediately frozen in liquid nitrogen. Whole-cell extracts for histones were prepared by trichloroacetic acid (TCA) extraction as described previously (3). 2 μl of crude protein extracts were spotted onto nitrocellulose membranes (Bio-Rad) using narrow-mouth pipette tips. Non-specific binding sites were blocked by soaking in 5% BSA in TBS-T for 30 min at room temperature. The membranes were incubated with the rabbit anti-H3K56ac primary antibody (1:1000) dissolved in BSA/TBS-T for 30 min at room temperature. After washing

the membranes three times with TBS-T (3x5 min each), the membranes were incubated with a secondary anti-rabbit antibody conjugated with HRP for 30 min at room temperature. After washing three times with TBS-T (3x5 min each) and once with TBS, the membranes were incubated with the ECL reagent for one minute and exposed to an X ray film in a dark room. Signal intensities were quantified using ImageJ.

DSB detection by Southern blot

Cells were harvested from meiotic time courses at the indicated time points. Genomic DNA was prepared as described in (3) and digested with appropriate restriction enzymes (indicated in the figures) according to (4). Digested DNA samples were run in 1% agarose gels prepared in 1X TBE at 2.3 V/cm for 16hr. DSBs were detected by Southern blot and indirect end-labeling with a ^{32}P-labeled DNA probe. Probes were designed to the termini of restriction fragments indicated in the cartoons and amplified by PCR. The hybridization signal was recorded by a phosphorimager (Typhoon, GE Healthcare) and quantified by ImageJ. DSBs ratios were expressed as the percent of radioactivity of the DSB fragment compared to the total radioactivity of the whole lane.

Chromatin immunoprecipitation and microarray (ChIP-on-Chip)

20 ml of sporulating yeast cells ($4x10^8$ cells) were harvested at the indicated time points in the sporulation medium, and samples were processed for ChIP and microarray hybridization as described (1). Immunoprecipitations were performed using 3 µl of rabbit polyclonal anti-Rfa1 antibody (provided by Steven Brill), and 3 µl of rabbit polyclonal anti-H3K56ac antibody (provided by Alain Verreault, Montreal), and 3 µl of rabbit polyclonal anti-H3Cter antibody (Abcam no. 1791). Raw histone H3K56ac microarray data (log_2 ratios) were normalized to histone H3 occupancy levels during meiosis (GSM274190 and GSM274191, (1)). The RPA microarray data were normalized by the background-based normalization method described previously (5). All microarray data are freely accessible and can be browsed in JBrowse using the following url: http://geneart.med.unideb.hu/pub/h3k56ac, login: h3k56ac; password: Mozaic4. Raw data are available at GEO (GSE37487).

Meiotic transcriptome analysis

mRNA was isolated from 20 ml of sporulating cultures at 0, 2, 4, 6 hs in SPM and used for cDNA synthesis (2 mg / time point) incorporating aminoallyl-dUTP (Fermentas). The 0 h sample was labelled with Cy3 (Amersam) while the 2/4/6 hs samples were coupled to Cy5 (Amersham) as described (1). Cy3 labelled cDNA was then hybridized together with Cy5-labelled samples to 15K transcriptomic microarrays (Agilent). Washes, scanning, data normalizations and analysis were performed as described (1,6). Two independent biological replicates were analyzed per timepoint. The analysis results can be found in Supplementary Table S1.

List of yeast strains used in this study

Strain ID	Genotype	Source
**NHY1210**	*Mat a /α, ho::hisG / ho::hisG, leu2::hisG / leu2::hisG, ura3(ΔSmaI-PstI) / ura3(ΔSmaI-PstI), HIS4::LEU2-(BamHI;+ori) / his4-X::LEU2-(NgoMIV; +ori)--URA3*	Neil Hunter lab
asf1Δ	*Mat a /α, ho::hisG / ho::hisG, leu2::hisG / leu2::hisG, ura3(ΔSmaI-PstI) / ura3(ΔSmaI-PstI), HIS4::LEU2-(BamHI;+ori) / his4-X::LEU2-(NgoMIV; +ori)--URA3, asf1Δ::KanMX / asf1Δ::KanMX*	This study
hst3/4Δ	*Mat a /α, ho::hisG / ho::hisG, leu2::hisG / leu2::hisG, ura3(ΔSmaI-PstI) / ura3(ΔSmaI-PstI), HIS4::LEU2-(BamHI;+ori) / his4-X::LEU2-(NgoMIV; +ori)--URA3, hst3Δ::KanMX / hst3Δ::KanMX, hst4Δ::HphMX / hst4Δ::HphMX*	This study
rtt109Δ	*Mat a /α, ho::hisG / ho::hisG, leu2::hisG / leu2::hisG, ura3(ΔSmaI-PstI) / ura3(ΔSmaI-PstI), HIS4::LEU2-(BamHI;+ori) / his4-X::LEU2-(NgoMIV; +ori)--URA3, trp1-1 / trp1-1, rtt109Δ::TRP1 / rtt109Δ::TRP1*	This study
**H3 (ctrl) *DMC1***	*Mat a /α, ho::hisG / ho::hisG, hht1::HphMX / hht1::HphMX, hht2::KanMX / hht2::KanMX, trp1-1 / trp1-1, his4 / his4, ura3 / ura3, pARS-CEN(HHT2, HHF2, TRP1)*	This study
**H3 (ctrl) *dmc1Δ***	*Mat a /α, ho::hisG / ho::hisG, dmc1::LEU2 / dmc1::LEU2, hht1::HphMX / hht1::HphMX, hht2::KanMX / hht2::KanMX, trp1-1 / trp1-1, his4 / his4, ura3 / ura3, pARS-CEN(HHT2, HHF2, TRP1)*	This study
**H3K56A *DMC1***	*Mat a /α, ho::hisG / ho::hisG, hht1::HphMX / hht1::HphMX, hht2::KanMX / hht2::KanMX, trp1-1 / trp1-1, his4 / his4, ura3 / ura3, pARS-CEN(hht2-K56A, HHF2, TRP1)*	This study
**H3K56A *dmc1Δ***	*Mat a /α, ho::hisG / ho::hisG, dmc1::LEU2 / dmc1::LEU2, hht1::HphMX / hht1::HphMX, hht2::KanMX / hht2::KanMX, trp1-1 / trp1-1, his4 / his4, ura3 / ura3, pARS-CEN(hht2-K56A, HHF2, TRP1)*	This study

Supplementary references

1. Borde V, Robine N, Lin W, Bonfils S, Geli V, Nicolas A. Histone H3 lysine 4 trimethylation marks meiotic recombination initiation sites . *EMBO J* (2009) **28**:99–111.

2. Blitzblau HG, Chan CS, Hochwagen A, Bell SP. Separation of DNA replication from the assembly of break-competent meiotic chromosomes. *PLoS Genet* (2012) **8**: doi:10.1371/journal.pgen.1002643

3. Acquaviva L, Székvölgyi L, Dichtl B, Dichtl SB, de La Roche Saint André C, Nicolas A, Géli V. The COMPASS subunit Spp1 links histone methylation to initiation of meiotic recombination. *Science* (2013) **339**:215–8. doi:10.1126/science.1225739

4. Murakami H, Borde V, Nicolas A, Keeney S. Gel electrophoresis assays for analyzing DNA double-strand breaks in Saccharomyces cerevisiae at various spatial resolutions. *Methods MolBiol* (2009) **557**:117–142.

5. Buhler C, Borde V, Lichten M. Mapping meiotic single-strand DNA reveals a new landscape of DNA double-strand breaks in Saccharomyces cerevisiae. *PLoS Biol* (2007) **5**:e324. doi:10.1371/journal.pbio.0050324

6. Sollier J, Lin W, Soustelle C, Suhre K, Nicolas A, Géli V, de La Roche Saint-André C. Set1 is required for meiotic S-phase onset, double-strand break formation and middle gene expression. *EMBO J* (2004) **23**:1957–67. doi:10.1038/sj.emboj.7600204
